# Kidney Response to the Spectrum of Diet-Induced Acid Stress

**DOI:** 10.3390/nu10050596

**Published:** 2018-05-11

**Authors:** Nimrit Goraya, Donald E. Wesson

**Affiliations:** 1 Baylor Scott & White Health Department of Internal Medicine, Temple, TX 76508, USA; Nimrit.Goraya@BSWHealth.org; 2 A&M Health Science Center College of Medicine, Temple, TX 76508, USA; 3 Baylor Scott & White Health Department of Internal Medicine, Dallas, TX 75210, USA; 4 A&M Health Science Center College of Medicine, Dallas, TX 75210, USA

**Keywords:** alkali, base, bicarbonate, chronic kidney disease, diet, protein

## Abstract

Chronic ingestion of the acid (H^+^)-producing diets that are typical of developed societies appears to pose a long-term threat to kidney health. Mechanisms employed by kidneys to excrete this high dietary H^+^ load appear to cause long-term kidney injury when deployed over many years. In addition, cumulative urine H^+^ excretion is less than the cumulative increment in dietary H^+^, consistent with H^+^ retention. This H^+^ retention associated with the described high dietary H^+^ worsens as the glomerular filtration rate (GFR) declines which further exacerbates kidney injury. Modest H^+^ retention does not measurably change plasma acid–base parameters but, nevertheless, causes kidney injury and might contribute to progressive nephropathy. Current clinical methods do not detect H^+^ retention in its early stages but the condition manifests as metabolic acidosis as it worsens, with progressive decline of the glomerular filtration rate. We discuss this spectrum of H^+^ injury, which we characterize as “H^+^ stress”, and the emerging evidence that high dietary H^+^ constitutes a threat to long-term kidney health.

## 1. Introduction

Optimal cell and tissue function requires maintenance of the “free” body fluid hydrogen ion (H^+^) concentration ([H^+^]) within a relatively narrow, slightly alkaline range compared to pure or “neutral” H_2_O: [H^+^] = 100 nM or 100 × 10^−9^ M = 10^−7^ M = 10^−7^ moles/liter. Because the pH of an aqueous solution is its negative log in moles/liter, pure or “neutral” H_2_O has a pH of 7. “Free” H^+^ appears to be the component of body H^+^ that mediates its physiological and pathophysiological actions and so its measurement is of interest to clinicians. Nevertheless, most body H^+^ is bound to other moieties (i.e., is “buffered”) and so is not “free” in solution. Although buffered H^+^ appears to have less direct physiological and/or pathophysiological actions, buffering of H^+^ can cause tissue damage [1], and the degree of H^+^-buffering might initiate signaling of downstream actions such as increased kidney acidification [2].

Under normal steady-state conditions, multiple and redundant systems maintain human plasma [H^+^] within 35–45 nM (pH 7.46–7.35), with “normal” considered to be [H^+^] = 40 nM or pH = 7.40. Clinicians have access to plasma as their “window” into an individual’s acid–base status but other compartments, like the interstitial compartment of extracellular fluid, are also affected by challenges to the systemic acid–base balance and likely play important contributory roles to body responses to these challenges. Acidosis is a process characterized by a net gain of H^+^ (caused by gain of H^+^ or by loss of buffer, most commonly HCO_3_), whereas a net loss of H^+^ (caused by gain of base, typically HCO_3_, or loss of H^+^) is called alkalosis. Despite daily acid–base challenges from the diet and metabolic processes, the body’s buffer systems and responsive changes to kidney acidification are typically successful in maintaining plasma [H^+^] within the normal range described. Laboratory techniques to be discussed provide insight into how these challenges affect both the plasma and interstitial fluid compartments.

Some chronic conditions associated with a decreased glomerular filtration rate (GFR), particularly chronic kidney disease (CKD), are associated with the accumulation of H^+^ characterized by an initial decrease in plasma bicarbonate concentration ([HCO_3_]), leading to metabolic acidosis. Deviations from normal toward the acid spectrum of acid–base balance are associated with premature death compared to patients with a normal acid–base status. For example, patients with non-dialysis-dependent CKD and metabolic acidosis have increased rates of mortality compared to CKD patients without metabolic acidosis [3,4]. Furthermore, mortality and adverse cardiovascular outcomes increase in CKD patients as plasma total CO_2_ (TCO_2_, nearly identical to plasma [HCO_3_]) decreases due to metabolic acidosis, and this increased risk extends into the normal range for plasma TCO_2_ [4]. Because diets typical of developed societies are H^+^-producing because of the predominance of H^+^-producing animal proteins compared to base-producing plant proteins [5], residents in such societies are chronically challenged with dietary H^+^. This “fixed” (sometimes called “non-volatile” to distinguish it from acidosis due to accumulation of carbon dioxide gas which is called respiratory acidosis) H^+^ is excreted predominantly by the kidneys. Mechanisms employed by the kidneys to excrete the ingested H^+^ yield the short-term benefit of enhanced H^+^ excretion but at the apparent long-term detriment of chronic kidney injury [2].

In the current study, we consider the consequences of this chronic dietary H^+^ challenge and the potential for kidney injury according to a spectrum of what we will call “H^+^ stress”. We characterize components of this spectrum according to the kidney and plasma acid–base context in which this chronic dietary H^+^ challenge occurs, under the following conditions: (1) high dietary H^+^ in the setting of normal kidney function as measured by the glomerular filtration rate (GFR) but without metabolic acidosis; (2) reduced GFR but no metabolic acidosis; and (3) reduced GFR with metabolic acidosis.

## 2. The Daily H^+^ Challenge

The body defends against H^+^ challenges to the acid–base balance by (1) excreting volatile, H^+^-producing CO_2_ gas through the lungs (about 15,000 mEq/day); (2) liver metabolism of base-producing food components (many plant-based foods, including most fruits and vegetables) and some organic products of metabolism (e.g., lactic acid) to yield HCO_3_; and (3) urine excretion of “fixed” acid (about 1 mEq/Kg/body wt/day) [5] from the liver metabolism of H^+^-producing food components (like animal-based protein) and some endogenous metabolic products. This yields the regeneration of new HCO_3_ by the kidney which restores HCO_3_ that had been titrated by added H^+^. Dietary sodium chloride (NaCl) also increases net endogenous acid production [6].

Diets typical of developed societies are acid-producing because of the preponderance of acid-producing compared to base-producing food components [7]. Typical developed society diets contain more animal-compared to plant-based food components. Animal-based food components contain more protein, and the contained protein has more of the sulfur-containing amino acids, methionine and cysteine which, when metabolized, yield sulfuric acid. Most fruits and vegetables also contain potassium salts of organic acids which, when metabolized, yield HCO_3_. Fresh animal-based foods contain various amounts of NaCl whereas fresh fruits and vegetables are very low in both Na^+^ and Cl^-^. Processed foods from animal and plant sources constitute an increasing proportion of developed society diets, and such foods have added NaCl. Dietary fats and sugars contribute comparatively less to net acid production when completely metabolized. The greater quantity of animal-based compared to plant-based foods, of processed compared to fresh foods, and the high NaCl content all contribute to the acid-producing nature of diets typical of developed societies.

Reducing the daily H^+^ challenge involves limiting food components that yield H^+^ when metabolized, such as animal proteins, dairy products, most grains, and lentils [8]. As mentioned, limiting dietary NaCl also reduces net endogenous acid production [6]. On the other hand, increasing the intake of base-producing foods also counteracts dietary acid production but the amount of base-producing potential varies among foods. Foods with high base-producing potential include many fruits and vegetables, including raisins, cruciferous vegetables (e.g., broccoli, brussel sprouts, cabbage), leafy greens (kale, spinach, lettuce, collard greens), and soy protein [8]. Substituting base-producing for acid-producing foods is a particularly effective strategy for dietary H^+^ reduction [9].

## 3. Maintenance of Normal Acid–Base Homeostasis

The maintenance of a normal, steady-state systemic acid–base status involves the elegant integration of physiological mechanisms such as extra- and intracellular buffering processes and collaborative actions of a number of organs, including the kidneys, liver, lungs, gastrointestinal tract, and skeleton. Recognizing the normal presence of these defense mechanisms reminds clinicians that when a disturbance of acid–base balance becomes evident, in part through changes in plasma acid–base parameters, the change in plasma [H^+^] reflects an exceeded capacity of (1) body buffers to prevent a change in plasma [H^+^]; and/or (2) organ(s) that ordinarily defend against changes in [H^+^] or restore it to normal, at least temporarily. For the remainder of our discussion, we will focus on the response to added H^+^ and not to its removal or to addition of HCO_3_.

Body systems can ameliorate the effect of added H^+^ to increase plasma [H^+^] (i.e., decrease pH) and decrease plasma [HCO_3_] by (1) employing the HCO_3_/H_2_CO_3_ buffer system; (2) binding the added H^+^ to non-HCO_3_ buffers in both the extra- and intracellular fluid; (3) sequestering added H^+^ in a non-plasma fluid compartment like interstitial fluid; (4) H^+^ excretion from the body, predominantly by the kidney through the urine; and/or (5) neutralization by endogenous or exogenous organic products.

### 3.1. Buffers

*HCO_3_/H_2_CO_3_ buffer system.* Adding H^+^ to body fluids containing HCO_3_ leads to the following process:

H^+^ + HCO_3_ → H_2_CO_3_ → H_2_O + CO_2_↑ (CO_2_ gas is excreted from the body by the lungs)

Consequently, added H^+^ is effectively removed from plasma as CO_2_ gas that would otherwise yield H^+^ (in a reversal of the above equation) if it were to accumulate, avoiding the other type of acidosis, respiratory acidosis. This rapidly responsive system works well to minimize the increase in [H^+^] (i.e., decrease in pH) that would otherwise occur in the absence of this elegant system. The price paid is a reduction in plasma [HCO_3_] that must be regenerated through H^+^ excretion by the kidneys (see below).

*Non-HCO_3_ buffers.* It is “free” H^+^ that determines the acid–base effect on cell and tissue function. Binding H^+^ to buffers takes it out of solution and greatly diminishes its untoward effects. The major non-HCO_3_ extracellular buffers are hemoglobin and albumin, whereas phosphate ion and anionic proteins are the major non-HCO_3_ intracellular buffers. Patients with CKD commonly have decreased plasma levels of hemoglobin and albumin, compromising the extracellular buffering capacity. Quantitatively, most H^+^ binding to non-HCO_3_ buffers occurs intracellularly [10]. Bone calcium carbonate and dibasic phosphate are important buffers for both acute and chronic metabolic acidosis in patients [1]. Experimental animals given an increment in dietary H^+^ that increased steady-state urine net acid excretion (NAE) but did not change steady-state plasma acid–base parameters, including plasma [HCO_3_], nevertheless had a decreased plasma blood base excess [11], consistent with increased titration of non-HCO_3_ buffers. In contrast, animals given aldosterone and Na_2_SO_4_ to increase urine NAE without an increment in dietary H^+^ did not have a decreased blood base excess [11], supporting the importance of dietary H^+^, and not ongoing enhanced urine NAE, per se, as the factor that led to the titration of non-HCO_3_ buffers.

### 3.2. H^+^ Sequestration in Interstitial Fluid

The kidney interstitium occupies strategic space between the peritubular capillaries and kidney tubules, facilitating potential chemical and cytokine communication between the plasma and tubule fluid [12]. Microdialysis of the kidney cortex allows the assessment of the chemical and cytokine content of the kidney interstitium [13]. Experimental animals given dietary H^+^ that increased steady-state urine NAE but did not change steady-state plasma acid–base parameters, including plasma [HCO_3_], nevertheless had an increased kidney cortical interstitial fluid H^+^ content, assessed with microdialysis [11]. This so-called “H^+^ retention” in response to an increment in dietary H^+^ was greater in animals with reduced GFR [14,15,16,17], even when plasma acid–base parameters were not different from those of control subjects [15,16,17]. In addition to the kidney cortex, skeletal muscle had H^+^ retention [15], supporting that this was a systemic phenomenon, at least in the setting of reduced GFR. Importantly, H^+^ retention persisted until the dietary H^+^ increment discontinued or was counterbalanced by added dietary alkali [11,17]. Excess body H^+^ sequestered from plasma in interstitial fluid might have the benefit of minimizing H^+^ accumulation in plasma along with its possible untoward consequences, while simultaneously stimulating basolateral kidney tubule mechanisms that enhance H^+^ excretion [18,19,20,21].

As mentioned, the diets of individuals in developed societies are typically H^+^-producing so their “normal” plasma acid–base parameters in this setting reflect their high H^+^-producing diet. Nevertheless, even large increments in dietary H^+^ lead to little to no measurable changes in plasma acid–base parameters, and the small changes that occur typically do so within the normal ranges of clinical laboratories [22]. These data show the effectiveness of body buffers, H^+^ sequestration, including in interstitial fluid, and kidney excretory capacity to maintain plasma [H^+^]/[HCO_3_], but also highlight the challenge for clinicians in gauging dietary H^+^ from plasma acid–base parameters. Patients with normal GFR given an increment in dietary H^+^ have cumulative urine H^+^ excretion less than the cumulative increment in dietary H^+^, consistent with H^+^ retention [1]. Other studies support the presence of H^+^ retention in CKD patients with reduced estimated glomerular filtration rate (eGFR) [23,24,25,26], even in the absence of metabolic acidosis [23,25,26], which is greater in magnitude in those with lower eGFR [26]. On the other hand, CKD patients with reduced GFR can have greater increases in [H^+^] and greater decreases in [HCO_3_] in response to the same increment in dietary H^+^, even developing metabolic acidosis at levels of dietary H^+^ that do not cause metabolic acidosis in patients with higher GFR [27]. Consequently, clinicians are more likely to recognize metabolic acidosis in patients with lower compared to higher GFR.

**Figure 1:**
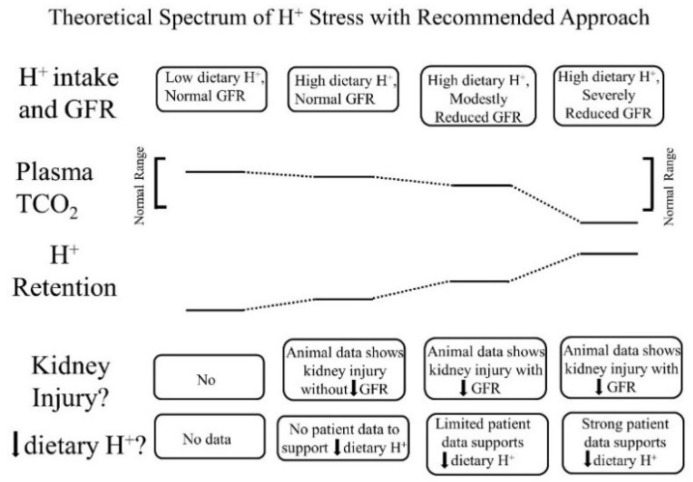
Theoretical spectrum of acid (H^+^) stress with a recommended approach for each stage. The top horizontal line of boxes characterizes four progressive stages of H^+^ retention according to a combination of dietary H^+^ intake and remaining glomerular filtration rate (GFR). The next two horizontal lines display figuratively a comparison between plasma total CO_2_ (TCO_2_) and tissue H^+^ retention among the four stages. The last two horizontal lines of boxes describe the existence, or not, of kidney injury in the four stages and whether individuals at each indicated stage should reduce dietary H^+^.

In addition to apparently reflecting systemic acid–base status, the interstitial fluid compartment appears to also reflect the systemic status of other kidney-regulated phenomenon, including sodium levels. Previous studies in patients have reported a direct relationship between the extracellular fluid volume and both the interstitial fluid volume and pressure, and each of the latter were higher in patients with reduced GFR [28]. These data support a continuum of “H^+^ stress” in which H^+^ retention is an earlier manifestation, and metabolic acidosis is a later manifestation (Figure 1).

### 3.3. Urine H^+^ Excretion

*Importance of urine buffers in kidney H^+^ excretion.* The kidney is the main contributor to the excretion of metabolically-produced, fixed H^+^. Diets of individuals in developed societies typically produce the equivalent of 60 to 100 mmoles of H^+^ daily when metabolized. To excrete 100 mmoles of H^+^ in the typical daily urine volume of 1 liter would require excreted urine to have a [H^+^] = 100 mmoles/liter = 100 × 10^−3^ M = 10^−1^ M = pH of 1.0 (remember that pH is the negative log of the [H^+^] in moles/liter). Because humans are unable to reduce urine pH below 4.0 (=[H^+^] of 10^−4^ M) which is equal to [H^+^] of 0.1 mM = 0.1 mmoles/liter, to excrete 100 mmoles of H^+^ in urine with pH 4.0 would require 100 mmoles/0.1 mmoles/liter = 1000 liters of urine. Hence, kidneys excrete fixed H^+^ predominantly as H^+^ bound to buffers, not as free H^+^ in solution. Quantitatively, ammonium [NH_4_^+^ from NH_3_ + H^+^] is the most important urine buffer, particularly in response to increments in dietary H^+^, followed by “titratable acidity”, most of the latter being phosphate (HPO_4_ = → H_2_PO4^−^).

*Afferent signal for H^+^ secretion.* The signal(s) that tell the kidneys to increase overall urine H^+^ excretion in response to (1) an increment in dietary H^+^ with normal GFR but in the absence of metabolic acidosis; (2) a reduced functioning nephron capacity (reduced GFR) in the setting of a standard H^+^-producing diet in individuals from developed societies (which requires greater per nephron H^+^ excretion to achieve the same overall H^+^ excretion as when there was a full contingent of functioning nephrons) but no metabolic acidosis; or (3) metabolic acidosis, whatever the underlying GFR. Nevertheless, the interstitial fluid compartment appears to be an appropriate one from which an afferent signal might initiate a change in kidney tubule acidification in response to the three settings described. Unlike plasma, interstitial fluid is comparatively protein-free, making its [H^+^] very sensitive to H^+^ addition or removal, unlike the highly buffered plasma compartment. Consequently, even small amounts of H^+^ added to the interstitial compartment will cause comparatively large changes in [H^+^]. This arrangement makes the interstitial fluid compartment a better one in which to house the “acidstat” than plasma, to monitor the acid–base status and respond with a signal to change tubule acidification. If plasma was the main or exclusive compartment housing the “acidstat”, wide swings in free [H^+^] would have potentially catastrophic consequences to physiological function. Microdialysis reveals dramatic increases in interstitial [H^+^] induced by increments in dietary H^+^ which cause no or comparably small changes in plasma acid–base parameters [11,15,16,17]. We hypothesize that pH sensors, such as GPR4 [29], sense these [H^+^] changes in the interstitial fluid compartment. Other or additional signals might determine the degree of titration of body buffers [11].

*Efferent response leading to increased kidney tubule acidification.* Because metabolically-produced H^+^ consumes body fluid HCO_3_, that after being filtered through the kidney tubules must not only be reabsorbed (recovered), but new HCO_3_ must be regenerated. These two main kidney tasks are accomplished by H^+^ secretion from kidney tubule cells into the tubule lumen. When there is a high amount of HCO_3_ in the tubule lumen, as is the case in the proximal tubule, secreted H^+^ titrates luminal HCO_3_ to CO_2_ and H_2_O, as previously described, and CO_2_ gas diffuses into the cell and is reconstituted to HCO_3_ by the enzyme, carbonic anhydrase. Reconstituted HCO_3_ is transported across basolateral membranes of tubule cells to return to the systemic circulation. When the tubule HCO_3_ content is low, as in the distal nephron, most secreted H^+^ titrates non-HCO_3_ buffers that are excreted in the urine, mostly as NH_4_^+^ and titratable acids. The exit of secreted H^+^ from the body as described allows HCO_3_ to be regenerated. Overall, the process is called NAE and is equal in quantity to HCO_3_ regeneration. Increments in NAE promoted by increments in dietary H^+^ intake are mediated mostly by an increase in urine NH_4_^+^ excretion with smaller contributions from increased titratable acidity and decreased urine excretion of HCO_3_ [30]. Most CKD patients with reduced GFR can effect steady-state H^+^ excretion sufficiently to avoid progressive metabolic acidosis while eating diets typical of developed societies [31,32]. More advanced GFR reduction in CKD patients, however, is associated with reduced urine NH_4_^+^ excretion [25] which likely contributes to an individual’s progression along the H^+^ stress spectrum from worsening H^+^ retention to, eventually, metabolic acidosis.

### 3.4. Kidney Cytokine Response to Systemic H^+^ Challenge

*Dietary H^+^ increment with normal GFR.* An increment in dietary H^+^, whether through mineral H^+^ [18,33] or H^+^-producing dietary protein [19,20,21] increases kidney levels of endothelin-1 (ET-1) in experimental animals. These higher kidney levels of ET-1 have the short-term physiological benefit of enhancing kidney tubule acidification in response to this H^+^ challenge [33] but carry the long-term pathophysiologic detriment of increased kidney interstitial fibrosis [34] which is a component of progressive nephropathy [35]. In addition, an increment in dietary H^+^ is associated with increased kidney levels of aldosterone in animals with normal GFR [20], and aldosterone also causes kidney fibrosis [36]. Increased dietary H^+^ in animals with normal GFR is associated with the manifestation of kidney due to glomerulosclerosis and tubulo-interstitial injury but no GFR decrease [35].

*Baseline dietary H^+^ in subjects with reduced GFR but without metabolic acidosis.* Most CKD patients with reduced eGFR have remaining eGFR sufficient to avoid metabolic acidosis [37,38], yet many such patients have progressive eGFR decline [39] and in some, dietary H^+^ reduction reduces kidney injury and slows their rate of eGFR decline [40]. The most common experimental model used to study CKD is rats with 5/6 nephrectomy but GFR in such animals is low enough to be associated with metabolic acidosis [14]. These animals have progressive nephropathy that is ameliorated by dietary H^+^ reduction [41]. On the other hand, animals with 2/3 nephrectomy have reduced GFR but no metabolic acidosis, yet also have progressive GFR decline that is ameliorated by dietary H^+^ reduction mediated by a reduction in their associated H^+^ retention [15,42,43]. Animals with 2/3 nephrectomy have higher than control baseline kidney levels of ET-1 [42], aldosterone [42], and angiotensin II [43], all of which contribute to the enhanced kidney tubule acidification observed in this model [16,18]. Each of these cytokines is associated with progressive nephropathy [42,43], and the amelioration of progressive nephropathy induced by dietary H^+^ reduction in this model is associated with reduced levels of each cytokine [15,42,43]. In addition, receptor antagonists for each cytokine also ameliorate nephropathy progression [42,43], further supporting a role for each cytokine in the progressive nephropathy of this model.

Studies in CKD patients also support that H^+^-induced increased levels of these cytokines contribute to progressive nephropathy. For example, CKD patients with reduced eGFR but no metabolic acidosis had H^+^ retention that was associated with increased urine excretion of ET-1 and aldosterone, each of which were decreased by dietary H^+^ reduction [23]. In addition, dietary H^+^ reduction in similar patients fitting these characteristics reduced urine ET-1 excretion [40], reduced urine indices of kidney injury [44], and slowed the rate of eGFR decline [40].

*Baseline dietary H^+^ in subjects with reduced GFR and metabolic acidosis.* Animals in the 5/6 nephrectomy model of CKD show severely decreased GFR and metabolic acidosis with progressive nephropathy that is associated with increased baseline kidney levels of ET-1 [41]. Endothelin receptor antagonists and dietary H^+^ reduction with improvement of the underlying metabolic acidosis each ameliorate nephropathy progression in this model [41]. In addition, the ameliorated nephropathy progression due to dietary H^+^ reduction is associated with reduced kidney levels of ET-1 [41]. Other investigators have shown the importance of dietary H^+^-induced augmented kidney levels of NH_3_/NH_4_^+^ that stimulate complement-mediated kidney injury in the progressive nephropathy of this model [45]. Dietary H^+^ reduction has been shown to decrease NH_3_/NH_4_^+^ kidney levels and ameliorate nephropathy progression [45].

Dietary H^+^ reduction in CKD patients with reduced eGFR and metabolic acidosis reduces urine excretion of ET-1 and indices of kidney injury [46] and slows eGFR decline [46,47]. Patients in two studies had metabolic acidosis with plasma TCO_2_ < 22 mM and so fit the treatment criteria for metabolic acidosis in CKD with dietary H^+^ reduction based on the current guidelines [48]. On the other hand, dietary H^+^ reduction in CKD patients with reduced eGFR and metabolic acidosis but with plasma TCO_2_ above that for which current guidelines recommend treatment (22–24 mM) also slowed their rate of eGFR decline [49]. These latter CKD patients had increased urine excretion of angiotensinogen, an index of kidney angiotensin II levels [49], consistent with increased kidney levels of angiotensin II. Dietary H^+^ reduction that ameliorated eGFR decline in these patients also reduced urine excretion of angiotensinogen [49]. The latter association of angiotensin II with nephropathy progression supports the well-documented kidney-protective benefits of receptor angiotensin II receptor antagonists in CKD patients [50].

Together, these data support the idea that the kidney mechanisms employed to achieve the short-term benefit of enhanced urine H^+^ excretion in response to H^+^ stress carry the long-term detriment of kidney injury with the risk for nephropathy progression with GFR decline. Kidney injury in response to the H^+^ stress caused by the high H^+^ intake of diets typical in developed societies appears to be greater in the setting of reduced GFR and even more so when the reduced GFR is accompanied by metabolic acidosis.

## 4. Other Organ Contributors to Systemic Acid–Base Status

*Liver.* The metabolism of ingested amino acids by the liver yields H^+^, HCO_3_, or neither, depending on the nature of the amino acids ingested. As mentioned, diets typical of developed societies yield net H^+^ when metabolized [7] so kidneys of these individuals typically excrete H^+^, mostly as NH_4_^+^ and titratable acids, as previously described. The liver is a major provider of glutamine, the source of most NH_4_^+^ used for H^+^ excretion in the urine, as previously described.

*Lungs.* Cellular metabolism produces CO_2_ which, as indicated earlier, yields H^+^ when dissolved in aqueous solution, as follows:

CO_2_ + H_2_O → H_2_CO_3_ → HCO_3_ + H^+^.

The HCO_3_ might leave the extracellular fluid or be buffered in the extra- or intracellular fluid, leaving behind H^+^. Fortunately, extracellular fluid flowing through the lungs allows CO_2_ gas to be excreted rather than retained.

*Gut.* Dietary components which, when metabolized, impact the systemic acid–base status are absorbed through the gastrointestinal tract. These components include amino acids, as previously described, but also organic anions derived from bacterial metabolism of ingested carbohydrates, protein, and fat. Absorbed organic anions are potential bases because they can be metabolized by the liver to yield HCO_3_. Organic acids might also be retained in the gut to titrate HCO_3_ to H_2_O and CO_2_, thereby reducing the amount of gut HCO_3_ that might be absorbed into extracellular fluid. Finally, these organic acids might be excreted from the body in the stools, representing a loss of potential HCO_3_.

*Bone.* H^+^ in both acute and chronic metabolic acidosis is buffered by bone [51] leading to a loss of calcium and its accompanying base from bone [52].

## 5. General Dietetic Management Strategies for the Spectrum of H^+^ Stress

### Dietary H^+^ Reduction

Because diets of developed societies are typically H^+^-producing, dietary H^+^ reduction might be achieved by (1) limiting the intake of H^+^-producing dietary constituents like animal-based proteins; (2) adding base-producing, plant-sourced constituents, like fruits and vegetables; and/or (3) adding Na^+^-based alkali (avoiding potassium-based alkali for CKD patients with reduced GFR because of the reduced potassium-excreting capacity of some CKD patients), like NaHCO_3_ or Na^+^ citrate.

*Removing/limiting H^+^-producing dietary components.* The algebraic sum of acid-producing and base-producing food components yields the net effect of a given diet on net endogenous acid production. Consequently, dietary H^+^ reduction can be done by limiting the intake of H^+^-producing food components, including animal-based proteins [7], and limiting the intake of dietary NaCl [6].

*Adding base-producing dietary components.* Dietary H^+^ reduction might be done alternatively by adding or substituting base-producing foods, like base-producing fruits and vegetables, to the high H^+^ diets typical of developed societies. The acid contents of many foods have been published [8] and could be used to determine how much of which foods to prescribe to CKD patients. Adding base-producing fruits and vegetables reduced urine H^+^ excretion in CKD patients with reduced eGFR but no metabolic acidosis [44] and also improved metabolic acidosis in CKD patients whose eGFR was low enough to be associated with metabolic acidosis [49,53,54].

Base-producing fruits and vegetables prescribed in amounts equivalent to 50% of their calculated dietary H^+^ load amounted to adding 2 to 4 cups of these foods daily, depending on the particular fruit or vegetable [44,49,53]. All had eGFR >30 mL/min/1.73 m^2^ and were carefully selected to be at very low risk for hyperkalemia in response to the increased potassium load that accompanies fruits and vegetables. Therefore, clinicians should use caution when considering prescribing fruits and vegetables to CKD patients, particularly those with very low GFR.

*Na^+^-based alkali therapies.* Sodium bicarbonate (NaHCO_3_) is the common alkali salt used to reduce dietary H^+^ because it is effective, relatively well-tolerated, widely available, and inexpensive. Potassium bicarbonate is used less commonly, except in patients who require substantial HCO_3_ replacement (like those with proximal renal tubular acidosis) that is associated with large K^+^ losses in response to treatment. Potassium bicarbonate should be avoided in patients with very low GFR (< 25% of normal) because of the risk for potassium retention with hyperkalemia. Because citrate is metabolized to yield HCO_3_, Na^+^ citrate is often used in patients unable to tolerate NaHCO_3_. The use of Na^+^ citrate is limited by its unpleasant taste, comparatively high expense, and because it promotes gastric aluminum absorption [55]. Consequently, NaHCO_3_ is the Na^+^-based alkali salt that is most commonly used.

## 6. Considerations Regarding Management of the Spectrum of H^+^ Stress

- *Low dietary H^+^ intake, normal GFR:* Animals ingesting a plant protein, base-producing diet have low kidney levels of endothelin [35], angiotensin II [16], and aldosterone [42], likely because of their reduced need to increase H^+^ excretion compared to animals eating an animal protein, H^+^-producing diet. Because the kidney levels of these substances are low, comparably little to no kidney injury occurs [35]. These animal data support the lack of need for additional dietary H^+^ reduction for analogous patients fitting this construct.
- *High dietary H^+^ intake, normal GFR:* Such animals achieve steady-state H^+^ excretion which avoids progressive H^+^ retention, yet they have steady-state H^+^ retention [11,17] without any significant change in plasma TCO_2_ [11,17,18]. These animals also have increased levels of endothelin [18], angiotensin II [16], and aldosterone [42], each of which help to mediate increased H^+^ excretion in response to this increment in dietary H^+^. Despite increased kidney levels of these substances, 96 weeks of these H^+^-producing diets in animals did not decrease GFR but did increase kidney tubulo-interstitial fibrosis (TIF) [35], a feature of progressive nephropathy. High H^+^ diets might decrease serum [HCO_3_]/pH slightly, but such small decreases remain within normal limits for clinical laboratories and so would not be evident clinically [22]. Although epidemiological studies support the idea that high H^+^ diets increase the risk of developing CKD [56] or type 2 diabetes [57], there are no published interventional data examining the long-term effect of high dietary H^+^ on kidney or other organ function in patients with baseline normal GFR to inform a management recommendation. That being said, such patients would theoretically benefit from reducing their high dietary H^+^ content.
- *High dietary H^+^ intake, modestly decreased GFR, but no metabolic acidosis based on plasma acid–base parameters:* Such animal CKD models have H^+^ retention yet no significant decrease in plasma TCO_2_ [15,16,17,42]. They also have increased kidney levels of endothelin [42], angiotensin II [16,43], and aldosterone [42], and they have progressive GFR decline characterized by TIF without dietary H^+^ reduction [16,42,43]. Analogous CKD patients with modestly reduced eGFR have H^+^ retention but with plasma TCO_2_, similar to comparable patients with normal eGFR [23,24] and have increased urine excretion of endothelin and aldosterone [23]. Small-scale interventional studies have shown that such CKD patients have progressive eGFR decline without dietary H^+^ reduction and that dietary H^+^ reduction reduces urine excretion of endothelin and aldosterone [23], reduces indices of kidney injury [44], and slows eGFR decline [40]. Larger scale studies are required to determine if dietary H^+^ reduction should be standard-of-care for CKD patients with modestly-reduced GFR but no metabolic acidosis based on serum acid-base parameters.
- *High dietary H^+^ intake, severely decreased GFR with metabolic acidosis:* Analogous animal CKD models fitting this construct have increased kidney levels of endothelin [14] with progressive GFR decline that is slowed by dietary H^+^ reduction [41]. Analogous CKD patients with severely reduced eGFR have increased urine endothelin excretion [46], and small-scale interventional studies have shown progressive GFR decline that is ameliorated by dietary H^+^ reduction [46,47,49]. Current guidelines recommend treatment with Na^+^-based oral alkali for amelioration of the disturbed bone and muscle metabolism caused by the metabolic acidosis of CKD [48] and, as mentioned, recent studies support the proposal that this intervention also slows eGFR decline. Other small-scale studies have provided support for the benefit of base-producing fruits and vegetables in treating metabolic acidosis in CKD [49,53], but larger scale studies must confirm these studies before such interventions, which carry the risk of hyperkalemia due to the high potassium content of plant-based foods, becomes a standard recommendation for treating the metabolic acidosis of CKD. These fruit and vegetable interventions, however, might be effective adjuncts to Na^+^-based alkali to improve metabolic acidosis, particularly given that the fruit and vegetable intervention has additional salutary effects like better blood pressure control [49,53].

## 7. Conclusions

The H^+^-producing character of diets typical of developed societies provides a consistent homeostatic challenge that requires the body’s defense mechanisms to ameliorate the untoward effects of added H^+^ and ideally, to eventually excrete it, the latter done primarily by the kidneys. Because the H^+^ challenge in developed societies is constant, these defense mechanisms are continuously “on”, and they include increased kidney levels of cytokines that provide the short-term benefit of promoting kidney tubule H^+^ secretion for urine H^+^ excretion but subject kidneys to long-term tubulo-interstitial injury with the risk of progressive GFR decline. The cumulative increment in urine H^+^ excretion is less than the accompanying increment in dietary H^+^, yielding a state of H^+^ retention that increases in magnitude as GFR declines. Body defense mechanisms can minimize decreases in plasma TCO_2_ and pH in the early stages of H^+^ retention, but as GFR continues to decline, the body’s H^+^ defense mechanisms become compromised, and/or dietary H^+^ increases, plasma TCO_2_ and pH decrease below lower ranges for clinical laboratories, and so, metabolic acidosis manifests, as depicted in Figure 1.

**Figure 2:**
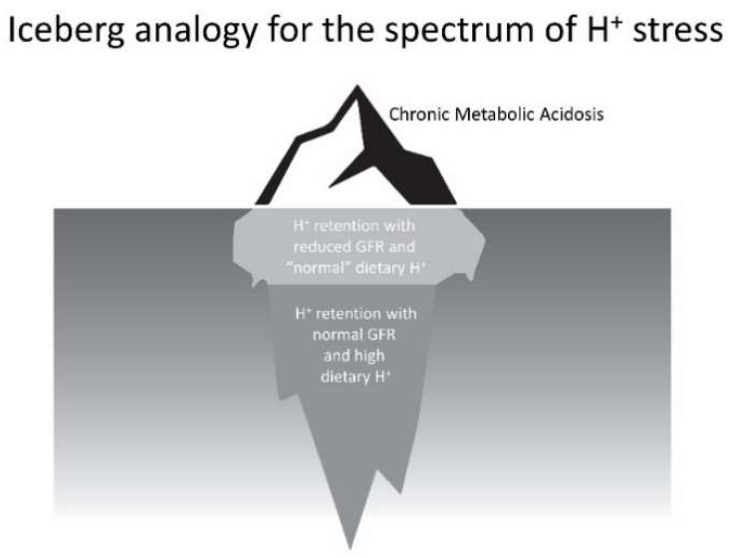
Figurative depiction of the stages of the theoretical spectrum of H^+^ stress using an iceberg analogy. Chronic metabolic acidosis, the portion above the water, is currently recognized by clinicians. The remaining indicated portions below water are not currently recognized by clinicians, yet emerging data support they are accompanied by threats to kidney health.

The scenario outlined describes the spectrum of the phenomenon that we characterize as H^+^ stress. Clinicians currently recognize only its most extreme manifestation, metabolic acidosis, as depicted above the water line in the iceberg analogy of Figure 2. Nevertheless, individuals shown below the water line, who have even modestly reduced eGFR and eat the H^+^-producing diets of developed societies, appear to have H^+^ retention, and small-scale studies have provided support that dietary H^+^ reduction is kidney-protective for them. More concerning, the greater number of individuals, who are also below the water line, with normal eGFR eating these H^+^-producing diets appear to be subject to long-term kidney injury and possibly increased risk for eventual eGFR decline. Future studies will determine if the latter two groups, currently not recognized by clinicians and who constitute earlier parts of the H^+^ stress spectrum, are candidates for dietary H^+^ reduction.
